# Risk of exacerbation in chronic obstructive pulmonary disease: a primary care retrospective cohort study

**DOI:** 10.1186/s12875-015-0387-6

**Published:** 2015-12-08

**Authors:** Josep Montserrat-Capdevila, Pere Godoy, Josep Ramon Marsal, Ferran Barbé, Leonardo Galván

**Affiliations:** Biomedical Research Institute (IRB) of Lleida, Lleida, Catalonia Spain; Health Department, Public Health Agency of Catalonia, Lleida, Catalonia Spain; Catalan Institute of Health (ICS), Mollerussa/Lleida, Catalonia Spain; Faculty of Medicine, University of Lleida, Lleida, Catalonia Spain; Lleida Research Support Unit, Primary Care Research Institute (IDIAP) Jordi Gol. Autonomous University of Barcelona, Lleida, Catalonia Spain; Cardiovascular Department, Epidemiology Unit, University Hospital Vall d’Hebron, Barcelona, Catalonia Spain; Pneumology Unit, University Hospital Arnau de Vilanova, Lleida, Catalonia Spain; Biomedical Research Centre Network for Respiratory Diseases (CIBERES), Madrid, Spain; Pharmacy Unit. Catalan Health Service, Lleida, Catalonia Spain

**Keywords:** Chronic obstructive pulmonary disease (COPD), Risk factors, Acute disease

## Abstract

**Background:**

The risk of exacerbation in chronic obstructive pulmonary disease (COPD) depends on the severity of disease and other less well known factors. Predictive models of exacerbation are more accurate than the forced expiratory volume in one second (FEV1). The objective was to design a model that predicts the risk of exacerbation in COPD.

**Methods:**

Retrospective cohort study with data from the electronic medical records of patients diagnosed with COPD in the province of Lleida (Spain). A total of 2501 patients were followed during 3 years. The dependent variable was acute exacerbation; independent variables were: clinical parameters, spirometry results, severity of disease, influenza and 23-valent pneumococcal immunisation, comorbidities, smoking and history of exacerbation. The association of these variables with disease exacerbation was measured by the adjusted odds ratio using a logistic regression model.

**Results:**

Mean age at the start of the study was 68.38 years (SD = 11.60) and 74.97 % patients were men; severity of disease was considered mild in 50.82 % of patients, moderate in 35.31 %, severe in 9.44 % and very severe in 4.44 %. During the three year study period up to 83.17 % of patients experienced at least one exacerbation. Predictive factors in the model were age, gender, previous exacerbations, influenza and 23-valent pneumococcal immunisations, number of previous visits to the General Practice and severity (GOLD), with an area under the ROC curve (AUROC) of 0.70.

**Conclusions:**

This model can identify patients at high risk of acute exacerbation. Preventive measures and modification of treatment in these high-risk patients would improve survival.

## Background

The global burden of chronic obstructive pulmonary disease (COPD) is high [1]. Some patients with COPD experience three episodes of acute exacerbation per year on average [2]. Most acute exacerbation episodes require medical attention, either in primary care or in hospital [3].

Predictive factors of exacerbation are still not completely understood [4–9]. However, acute exacerbations are known to accelerate the severity of disease, [3] contribute to irreversible decline of pulmonary function [10], have a negative impact on quality of life [11] and decrease survival [12]. The proportion of treatment failure in patients with acute exacerbations is high (20–40 %) [13] and it is associated with elevated health costs [14].

Traditionally, a decrease in forced expiratory volume in one second (FEV_1_) has been considered one of the main risk factors of exacerbation. However, FEV_1_ does not discriminate between stable periods of disease and acute exacerbations, and is less effective in the late stages of the disease [3]. Indeed, some patients with low FEV_1_ have never experienced acute exacerbations, and a higher FEV_1_ does not rule out the risk of exacerbation [15].

It is essential to consider COPD as a systemic disease rather than just pulmonary, since it is associated with symptoms in other organs and with comorbidities [16, 17] that can determine the risk of acute exacerbation. Consequently, new multifactorial predictive models for exacerbation are required [18]. The BODE index, which comprises Body Mass Index, airflow Obstruction, Dyspnoea and Exercise (distance walked in 6 min) is a predictive factor of mortality in COPD and a better predictive factor for acute exacerbation than FEV_1_ [19]. The study of Marin et al. showed that the BODE index was a better predictive method than FEV_1_ for acute exacerbation in patients with COPD [19]. However, the six minute walk test is not easily implemented in primary care. Other models have been proposed [20–22]. Nonetheless, new models with variables that can be obtained easily and quickly are needed.

Factors related to acute exacerbation are still poorly understood [5]. Some variables have been identified with risk of exacerbation. However, further studies are needed to prove the association of these factors with acute exacerbation and to validate models of prediction [23].

Factors such as the characteristics of the population, prevalence of risk factors, severity of COPD and aspects of the health system [3] result in a geographical variation in the incidence of exacerbation [24]. In consequence, predictive models should be adjusted to different settings.

The objective of this study was to determine predictive factors associated with exacerbation of COPD in a primary care cohort of patients in Catalonia (Spain) with a high percentage of mild and moderate severity during a three year follow up.

## Methods

A retrospective cohort study was carried out with 2501 patients with a COPD diagnosis from seven primary care centres of the Lleida Health Region (catchment population: 172,950). The criteria of inclusion were over 40 years of age with a diagnosis of COPD in 2010 in primary care electronic medical records and having a spirometry test in the last two years*.* GOLD criteria were followed for the diagnosis of COPD: [25] a post bronchodilator FEV_1_/FVC <0.7 when the patient is in the stable phase of the disease. For a patient to be included in the study, the electronic medical records must also contain the result of a spirometry test from the previous 2 years. The dependent variable was acute exacerbation of COPD, defined as an increase in dyspnoea, amount and purulence of sputum [26] that require treatment with systemic steroids and/or antibiotics (moderate exacerbations) or that require hospitalization (severe exacerbations) [27]. The referral hospitals were the University Hospital Arnau de Vilanova and the Hospital Santa Maria de Lleida. These were the two hospitals where the patients that required admission were referred. Exacerbations between November 2010 and October 2013 were computed. Data sources were the primary care electronic medical records (e-CAP) and the Health Department Pharmacy Unit Register. The following independent variables were collected at the beginning of the study: age, gender, spirometry results (FEV_1_/CVF, FVC, FEV_1_), comorbidities (heart failure, ischemic heart disease, diabetes, chronic kidney failure, atrial fibrillation and anaemia), history of smoking, 23-valent pneumococcal and influenza immunisations for the 2009/10 season, years since COPD was diagnosed, number of visits to the health centre and number of acute exacerbations in the year prior to the start of the study, and COPD severity according to the GOLD Guidelines.

This study was approved by the Clinical Research Ethics Committee of the Primary Care Research Institute (IDIAP) Jordi Gol of Barcelona (P14/022). This ethics committee determined that individual patient consent was not necessary due to the retrospective nature of the study. We also obtained the consent by management of all the seven primary care centres participating in the study (Lleida, Pla d’Urgell, Les Borges Blanques, Bellpuig, Tàrrega, Agramunt and Cervera) as well as the approval by the management of Primary Care of the Healthcare Region of Lleida. The Primary Care Management of the Catalan Institute of Health authorized permission to access the clinical records of the patients included in the study with the ultimate aim of gathering information concerning the variables of interest.

### Statistical analysis

A descriptive analysis of the data was performed. Quantitative variables were described using the mean and standard deviation and categorical variables using the absolute and relative frequencies. Hypothesis testing to evaluate the association of independent variables with the clinical outcome (COPD exacerbation) was performed using the Chi-square test for categorical variables and Student’s *t* test or Mann-Whitney’s *U* test for continuous variables. The crude odds ratio (OR) was calculated for each independent variable included in the model.

A derivation cohort (70 %) and a validation cohort (30 %) were randomly generated. The score was adjusted in the derivation cohort and the predicted probability, power and calibration were estimated from the 30 % included in the validation cohort. The score included all variables associated with an outcome with a *P* value <0.2. The risk score was adjusted for the 3-year period with a logistic regression analysis using automatic (forward and backward) variable selection algorithms. Selected variables were included in the model if the *P* value of the effect was <0.1. The characteristics of the calibration were evaluated by the Hosmer-Lemeshow test and the discriminatory power using the *c-* statistic of the area under the curve (AUC).

Statistical Interaction and quadratic effect were tested for all quantitative variables. Statistical significance was considered if *P*-value <0.05. All analyses were performed using the statistical package SPSS v15.0.

## Results

Mean age of the 2501 patients at the start of the study was 68.38 years (SD = 11.60) and 75 % were men. Severity of disease (GOLD) was mild in 50.82 % patients, moderate in 35.31 %, severe in 9.44 % and very severe in 4.44 %. The year prior to the start of the study a total of 20.23 % patients had experienced at least one episode of acute exacerbation and the average number of visits to the physician or nurse in primary care was 25. With regard to immunisation, 70.45 % had received the 23-valent pneumococcal vaccine and 71.41 % the influenza vaccine during the 2009/10 season (season prior to the start of the study).

Table 1 shows the characteristics of the patients in relation to acute exacerbations. Patients who experienced acute exacerbations were older, had attended the primary care centre more often during 2009, presented more comorbidities (heart failure, ischemic heart disease, diabetes, chronic kidney failure, atrial fibrillation and anaemia) and had experienced more acute exacerbations during the year prior to the start of the study. Up to 83.17 % of patients experienced at least one episode of acute exacerbation during the 3-year follow up. Patients with a higher severity of disease (GOLD grades III and IV) experienced higher rates of acute exacerbation (Table 2), and had higher rates of immunisation with the 23-valent pneumococcal and influenza vaccines during the 2009/2010 season (*p* <0.001).

**Table 1:** Characteristics of patients at the beginning of the study and description of variables associated with acute exacerbation during the 3-year follow-up

	Exacerbation (treatment during next 3y)			All (*n* = 2501)
	No (*n* = 421)	Yes (*n* = 2080)	*p*	
Age^a^	65.62 (11.90)	68.94 (11.50)	0.001	68.38 (11.60)
COPD History (years)^a^	2.86 (3.50)	3.69 (4.40)	0.001	3.55 (4.30)
Num. of visits (2009)^a^	18.01 (14.30)	25.96 (18.70)	0.001	24.63 (18.30)
FEV1	66.38 (23.20)	62.80 (24.40)	0.004	63.38 (24.30)
Gender (female)^b^	79 (18.76 %)	547 (26.30 %)	0.001	626 (25.03 %)
23-valent Pneumococcal^b^	242 (57.48 %)	1520 (73.08 %)	0.001	1762 (70.45 %)
2009 influenza vaccine^b^	242 (57.48 %)	1544 (74.23 %)	0.001	1786 (71.41 %)
Exacerbation 2009^b^	20 (4.75 %)	486 (23.37 %)	0.001	506 (20.23 %)
Number of comorbidities^c^	0.34 (0.70)	0.45 (0.70)	0.002	0.43 (0.70)
Smoking^b^	178 (42.28 %)	666 (32.02 %)	0.001	844 (33.75 %)
Gold (Severity)^b^			0.062	
Mild COPD	223 (52.97 %)	1048 (50.38 %)		1271 (50.82 %)
Moderate COPD	157 (37.29 %)	726 (34.90 %)		883 (35.31 %)
Severe COPD	27 (6.41 %)	209 (10.05 %)		236 (9.44 %)
Very Severe COPD	14 (3.33 %)	97 (4.66 %)		111 (4.44 %)
Hospital Admission^b^	62 (14.73 %)	750 (36.06 %)	0.001	812 (32.47 %)
Death^b^	39 (9.26 %)	275 (13.22 %)	0,025	314 (12.55 %)

^a^mean (SD): Mann–Whitney *U* test

^b^absolute and relative frequency. Chi-square test

^c^Number of comorbidities: Ischemic Heart Disease, Heart Failure, Diabetes, Anaemia, Atrial Fibrillation and Chronic Renal Failure

The statistical significance is fixed for *p*-value <0.05

**Table 2:** Immunisation and acute exacerbation in relation to severity of COPD

	Mild	Moderate	Severe	Very Severe	All	*p*
	(*n* = 1271)	(*n* = 883)	(*n* = 236)	(*n* = 111)	(*n* = 2501)	
23-valent Pneumococcal^a^	866 (68.14 %)	628 (71.12 %)	185 (78.39 %)	83 (74.77 %)	1762 (70.45 %)	0.002
2009 influenza vaccine^a^	880 (69.24 %)	636 (72.03 %)	183 (77.54 %)	87 (78.38 %)	1786 (71.41 %)	0.002
Exacerbation (treatment during next 3y or hospital admission)^a^	1048 (82.45 %)	726 (82.22 %)	209 (88.56 %)	97 (87.39 %)	2080 (83.17 %)	0.043

^a^Absolute and relative frequency. Chi-square test

**Fig. 1:**
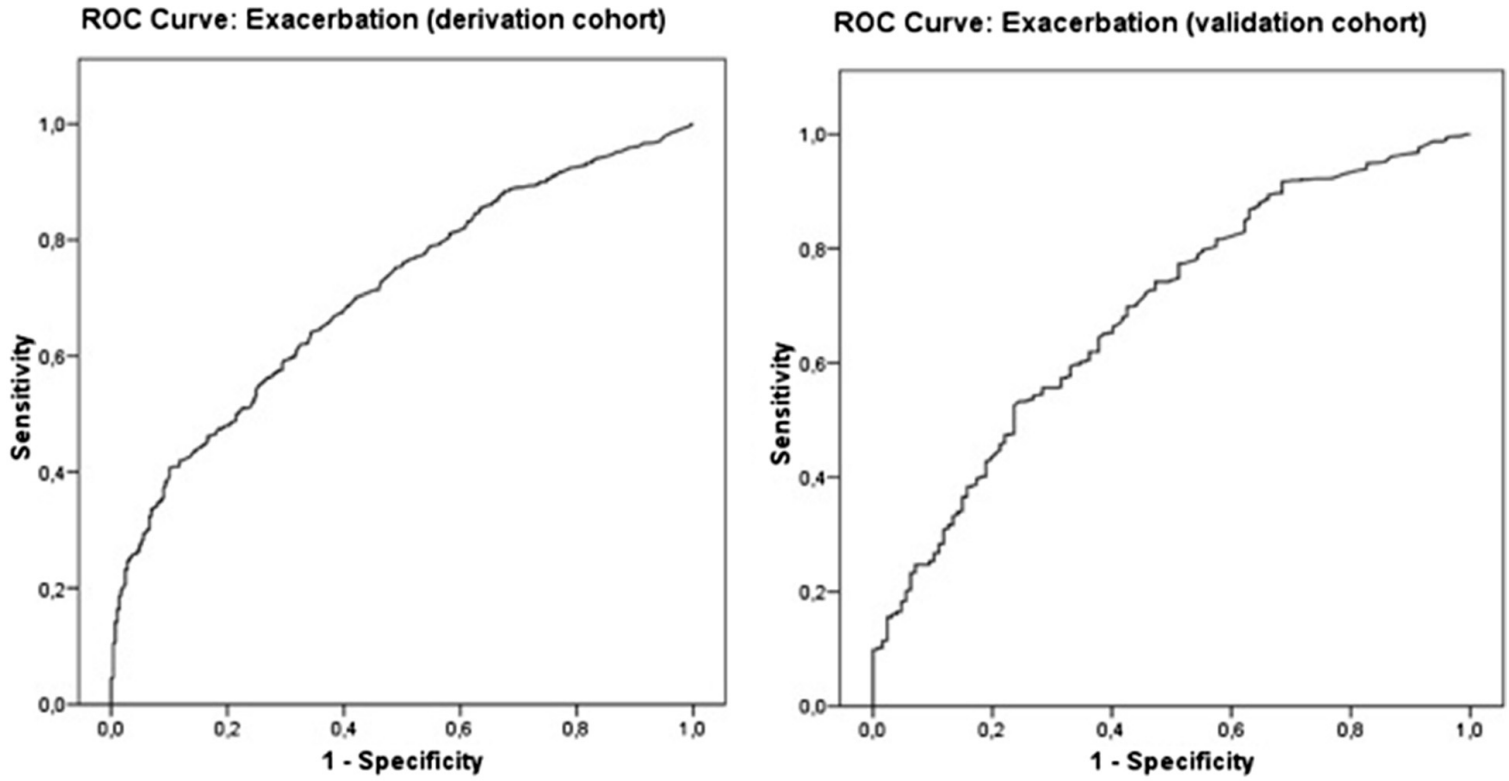
Exacerbation ROC curve after 3 years of follow up (validation and derivation cohorts)

The multivariate analysis (Table 3) shows that the following factors are significantly associated with COPD exacerbation: age (mean 68 years (_a_OR = 1.01), gender (woman) (_a_OR = 1.46), gender (woman) x age (mean 68 years (_a_OR = 0.96), previous exacerbations (_a_OR = 7.44), severe or very severe disease (GOLD), (_a_OR = 1.57), number of visits to the primary care centre (from 25 to 50 visits (_a_OR = 1.64); over 50 visits (_a_OR = 2.95) in relation to the standard (<25 visits), influenza vaccine (_a_OR = 2.00), 23-valent pneumococcal vaccine (_a_OR = 1.82), influenza x 23-valent pneumococcal vaccine (_a_OR = 0.46). Patients immunised with the pneumococcal and influenza vaccines had a 54 % lower risk of exacerbation. All these variables were significantly associated with COPD exacerbation, with a discriminatory power of 0.71. Fig. 1 shows the calibration and discriminatory power of the predictive model. The predictive factor more strongly associated with disease exacerbation was acute exacerbation during the year prior to the start of the study, which increased the risk of experiencing acute exacerbation by 7.44 during the following 3 years.

**Table 3:** Predictive factors of COPD exacerbation during the 3-year follow-up period

	Exacerbation (treatment in next 3y)		
	OR	95 % CI	p
Num. Of visits 2009 (Standard < 25v)			0.001
25–50	1.64	(1.17 – 2.29)	0.004
> 50	2.95	(1.25 – 6.94)	0.013
Gender (female)	1.46	(1.04 – 2.04)	0.028
Exacerbation	7.44	(3.77 – 14.70)	0.001
INT: Female * Age (mean 68y)	0.96	(0.93 – 0.99)	0.003
Age (mean 68y)	1.01	(1,00 – 1.03)	0.087
Gold severity grade (severe or very severe)	1.57	(1.01 – 2.45)	0.046
2009 influenza vaccine	2.00	(1.12 – 3.58)	0.019
23-valent Pneumococcal vaccine	1.82	(1.00 – 3.29)	0.049
INT: 2009 influenza vaccine * Pneumococcal	0.46	(0.20 - 1.02)	0.055
Derivation			
Hosmer & Lemeshow	1.10		0.997
AUC ROC	0.71	(0.68 – 0.74)	< 0.001
Validation			
Hosmer & Lemeshow	41.00		<0.001
AUC ROC	0.69	(0.64 – 0.74)	<0.001

*INT* Interaction

*OR* odds ratio

*OR was adjusted for: Num. of visits, gender, exacerbation, GOLD severity grade, influenza vaccine, 23-valent pneumococcal vaccine*

## Discussion

In this study, patients with at least one episode of exacerbation during the 3-year follow-up were older and more often men; they had a higher COPD severity grade (GOLD III and IV), they had experienced more acute exacerbation episodes during the year prior to the study and had attended the primary care centre more often due to a higher prevalence of comorbidities. Older patients experienced a higher rate of acute exacerbation episodes, probably because pulmonary function decreases with time and because of the accumulative effect of risk factors such as smoking. On the other hand, this study confirms the effectiveness of the influenza and 23-valent pneumococcal vaccines. Indeed, immunisation reduces risk practically by half. This model could be used in primary care visits to identify patients with a higher risk of exacerbation.

### Interpretation of results based on the literature

Other authors have researched similar predictive models for exacerbation, with slight differences. Miravitlles et al. [28] designed a model for exacerbation with the following variables: age, FEV_1_ (GOLD severity) and chronic sputum production, and obtained an area under the ROC curve (AUROC) of 0.601 . Our model also includes age and FEV_1_, though it achieves a higher discriminatory power. Motegi et al. [29] compared three predictive models of COPD exacerbation: the DOSE (dyspnoea, degree of airflow limitation, smoking and exacerbations); BODE (body mass index, degree of airflow limitation, dyspnoea and exacerbations); and ADO (age, dyspnoea, degree of airflow limitation) indices. These predictive models had a discriminatory power (ROC curve) of 0.75, 0.65 and 0.64, respectively. The authors concluded that the best model to predict future exacerbations in COPD patients was the DOSE index. Our model has a higher AUROC than those found by Motegi et al. for the BODE and ADO indices, and it also contains risk factors common to the three models: age, degree of airflow limitation and history of exacerbation. Make et al. [20] developed a score (SCOPEX) that included variables such as gender, number of COPD maintenance medications, number of exacerbations in the previous year, FEV1/FVC and number of daily short-acting bronchodilators for clinicians to predict exacerbations. Moberg et al. [21] used measures of exercise capacity and dyspnoea scores to develop the iBODE index as a predictor. While Schembri et al. [22] also developed a predictive model based on data collected from clinic visits on demography, spirometry, smoking history, dyspnoea score, BMI and other variables.

In our model, a history of exacerbation is the best predictive factor for exacerbation, followed by an age over 68. The study by Hurst et al. also concluded that a history of exacerbation during the previous year was the best predictive factor of acute exacerbation [27].

As in other studies, the male sex was a risk factor of exacerbation due to the smoking habits. However, other studies have reported a higher risk of exacerbation in females [30].

Our study further supports the evidence on the effectiveness of influenza and 23-valent pneumococcal immunisations in significantly reducing the risk of acute exacerbation [31, 32]. This study contributes a new predictive model of COPD exacerbation with variables that are easily and swiftly collected. The high discriminatory power makes this model adequate for use in primary care.

### Strengths and limitations of the study

The main strengths of the study are the length of the follow-up period (3 years) and characteristics of the cohort (the sample consists of a high percentage of patients with mild and moderate severity, unlike other studies, thus, our sample is more representative of the spectrum of the disease in the community. The primary care electronic medical records include all patients with a diagnosis of COPD in our region. In contrast, hospitals registers only include patients with the most severe forms of the disease. The study has also some limitations. The information was obtained from primary care electronic medical records, where some variables such as smoking and influenza and pneumococcal immunisations can be underreported. Some series like the UPLIFT studies found a prevalence of 29 % of smokers, [33] whereas another reports a prevalence as low as 4.4 % [6], much less than our study.

The study included patients with a diagnosis of COPD and with a spirometry result from 2 years prior to the start of the study. These inclusion criteria ensure that all patients were correctly diagnosed, but might exclude patients with COPD either because of underreporting or because they did not have a spirometry result in their electronic medical records. Such patients would probably be those with a mild disease. Also, patients were considered immunised against influenza or pneumococcal disease only when the immunisation was registered in the eCAP. When patients are immunised in private clinics it might not be adequately recorded. Some patients may give up smoking after the study and this change was not registered. Consequently, the effects of smoking and the protective effect of immunization might be underestimated. With regard to influenza immunisation, we only considered the 2009/10 seasonal immunisation. However, immunised patients tend to attend immunisations every year [34] and thus those immunised during the 2009/10 season were probably also immunised during the following seasons. Some variables studied in other models, such as weight/BMI, exercise/functional capacity or dyspnoea, were not investigated due to a lack of comprehensive information for all patients. Lastly, we must highlight that the predictors objectified in this study may vary depending on the geographical area of the study and the characteristics of health systems specific for each one, which is why more studies are needed in different health regions.

### Implications for future research and practice

Some risk factors might vary in different settings. Other variables should be investigated in other future models and in other places, like the number of medication [20], seasonality [35, 36], sleep disturbance[37] and exposure to pollutants [38, 39]. Our study reports risk factors of COPD exacerbation in a specific region of Spain. External validation of this predictive model is required before implementing it in other cohorts of patients with COPD.

## Conclusions

This predictive model of acute exacerbation in COPD can be easily and quickly implemented in primary care. Patients at high risk could be identified for preventive action, such as immunisation and for intensification of treatment. As a consequence, quality of life and survival rates in patients with COPD would improve.

## References

[1] Murray CJ, Lopez AD (1997). Alternative projections of mortality and disability by cause 1990–2020: Global Burden of Disease Study. Lancet.

[2] Wedzicha JA, Donaldson GC (2003). Exacerbations of chronic obstructive pulmonary disease. Respir Care.

[3] Franciosi LG, Page CP, Celli BR, Cazzola M, Walker MJ, Danhof M (2006). Markers of exacerbation severity in chronic obstructive pulmonary disease. Respir Res.

[4] Fabbri L, Beghé B, Caramori G, Papi A, Saetta M (1998). Similarities and discrepancies between exacerbations of asthma and chronic obstructive pulmonary disease. Thorax.

[5] Holgate ST (2007). Priorities for respiratory research in the UK. Thorax.

[6] Langsetmo L, Platt RW, Ernst P, Bourbeau J (2008). Underreporting exacerbation of chronic obstructive pulmonary disease in a longitudinal cohort. Am J Respir Crit Care Med.

[7] Baker CL, Zou KH, Su J (2013). Risk assessment of readmissions following an initial COPD-related hospitalization. Int J Chron Obstruct Pulmon Dis.

[8] Bertens LCM, Reitsma JB, Moons KGM, van Mourik Y, Lammers JWJ, Broekhuizen BDL (2013). Development and validation of a model to predict the risk of exacerbations in chronic obstructive pulmonary disease. Int J Chron Obstruct Pulmon Dis.

[9] Makita C, Nakamura T, Takada A, Takayama K, Suzuki M, Ishikawa Y (2014). Clinical outcomes and toxicity of proton beam therapy for advanced cholangiocarcinoma. Radiat Oncol.

[10] Seemungal TA, Donaldson GC, Bhowmik A, Jeffries DJ, Wedzicha JA (2000). Time course and recovery of exacerbations in patients with chronic obstructive pulmonary disease. Am J Respir Crit Care Med.

[11] Seemungal TA, Donaldson GC, Paul E a, Bestall JC, Jeffries DJ, Wedzicha JA (1998). Effect of exacerbation on quality of life in patients with chronic obstructive pulmonary disease. Am J Respir Crit Care Med.

[12] Burge S, Wedzicha JA. COPD exacerbations: definitions and classifications. Eur Respir J Suppl 2003;41:46 s - 53 s. 10.1183/09031936.03.00078002 12795331

[13] Donaldson GC, Seemungal T a R, Bhowmik a, Wedzicha J a (2002). Relationship between exacerbation frequency and lung function decline in chronic obstructive pulmonary disease. Thorax.

[14] Heart N (2004). Lung and Blood Institute. Morbidity and mortality chartbook on cardiovascular, lung and blood diseases.

[15] Mador MJ, Sethi S (2013). Systemic inflammation in predicting COPD exacerbations. JAMA.

[16] Oga T, Tsukino M, Hajiro T, Ikeda A, Nishimura K (2011). Predictive properties of different multidimensional staging systems in patients with chronic obstructive pulmonary disease. Int J Chron Obstruct Pulmon Dis.

[17] Wouters EFM (2002). Chronic obstructive pulmonary disease. 5: systemic effects of COPD. Thorax.

[18] van Dijk WD, van den Bemt L, van den Haak-Rongen S, Bischoff E, van Weel C, Veen JCCM (2011). Multidimensional prognostic indices for use in COPD patient care. A systematic review Respir Res.

[19] Marin JM, Carrizo SJ, Casanova C, Martinez-Camblor P, Soriano JB, Agusti AGN (2009). Prediction of risk of COPD exacerbations by the BODE index. Respir Med.

[20] Make BJ, Eriksson G, Calverley PM, Jenkins CR, Postma DS, Peterson S (2015). A score to predict short-term risk of COPD exacerbations (SCOPEX). Int J Chron Obstruct Pulmon Dis.

[21] Moberg M, Vestbo J, Martinez G, Williams JE a, Ladelund S, Lange P, et al. Validation of the i-BODE Index as a Predictor of hospitalization and Mortality in Patients with COPD Participating in Pulmonary Rehabilitation. COPD. 2013;1–7. 10.3109/15412555.2013.836171 24111845

[22] Schembri S, Anderson W, Morant S, Winter J, Thompson P, Pettitt D (2009). A predictive model of hospitalisation and death from chronic obstructive pulmonary disease. Respir Med.

[23] Al-ani S, Spigt M, Hofset P, Melbye H (2013). Predictors of exacerbations of asthma and COPD during one year in primary care. Fam Pract.

[24] Otero González I, Blanco Aparicio M, Montero Martínez C, Valiño López P, Verea Hernando H (2002). The epidemiology of COPD and asthma exacerbations in a general hospital. Arch Bronconeumol.

[25] Rabe KF, Hurd S, Anzueto A, Barnes PJ, Buist SA, Calverley P (2007). Global strategy for the diagnosis, management, and prevention of chronic obstructive pulmonary disease: GOLD executive summary. Am J Respir Crit Care Med.

[26] Sapey E, Stockley R a (2006). COPD exacerbations. 2: aetiology. Thorax.

[27] Hurst JR, Vestbo J, Anzueto A, Locantore N, Müllerova H, Tal-Singer R (2010). Susceptibility to exacerbation in chronic obstructive pulmonary disease. N Engl J Med.

[28] Miravitlles M, Guerrero T, Mayordomo C, Sánchez-Agudo L, Nicolau F, Segú JL (2000). Factors associated with increased risk of exacerbation and hospital admission in a cohort of ambulatory COPD patients: a multiple logistic regression analysis. The EOLO Study Group Respiration.

[29] Motegi T, Jones RC, Ishii T, Hattori K, Kusunoki Y, Furutate R (2013). A comparison of three multidimensional indices of COPD severity as predictors of future exacerbations. Int J Chron Obstruct Pulmon Dis.

[30] Kilic H, Kokturk N, Sari G, Cakır M (2015). Do females behave differently in COPD exacerbation?. Int J Chron Obstruct Pulmon Dis.

[31] Poole PJ, Chacko E, Wood-Baker RWB, Cates CJ (2006). Influenza vaccine for patients with chronic obstructive pulmonary disease. Cochrane Database Syst Rev.

[32] Viejo JLB (2009). Vaccination against pneumococcal infection in adults. Rev Esp Quimioter.

[33] Soler-Cataluña JJ, García MAM (2009). Impact of efficacy and mortality studies (TORCH and UPLIFT) in bronchodilator treatment of chronic obstructive pulmonary disease. Arch Bronconeumol.

[34] Montserrat-Capdevila J, Godoy P, Marsal J-R, Cruz I, Solanes M (2014). Effectiveness of influenza vaccination in preventing hospital admission due to exacerbations of chronic obstructive pulmonary disease. Enferm Infecc Microbiol Clin.

[35] Jenkins CR, Celli B, Anderson JA, Ferguson GT, Jones PW, Vestbo J, et al. Seasonality and determinants of moderate and severe COPD exacerbations in the TORCH study. Eur Respir J. 2012;38–45. 10.1183/09031936.00194610 21737561

[36] Donaldson GC, Goldring JJ, Wedzicha JA (2012). Influence of season on exacerbation characteristics in patients with COPD. Chest.

[37] Terada K, Muro S, Sato S, Ohara T, Haruna A, Marumo S (2008). Impact of gastro-oesophageal reflux disease symptoms on COPD exacerbation. Thorax.

[38] Ling SH, van Eeden SF. Particulate matter air pollution exposure: role in the development and exacerbation of chronic obstructive pulmonary disease. Int J Chron Obstruct Pulmon Dis. 2009;233–43. 10.2147/copd.s5098 PMC2699820 19554194

[39] Song Q, Christiani DC, Xiaorong W, Ren J (2014). The global contribution of outdoor air pollution to the incidence, prevalence, mortality and hospital admission for chronic obstructive pulmonary disease: a systematic review and meta-analysis. Int J Environ Res Public Health.

